# Case Report: Relapsing pleural effusions and coated aorta revealing Erdheim–Chester disease

**DOI:** 10.3389/fimmu.2025.1585541

**Published:** 2025-09-12

**Authors:** Tiépé Rokia Ouattara, Théo Pezel, Gwenael Lorillon, Aïcha Kante, Peggy Reiner, Aurélie Le Gal, Marine Lefèvre, Thibault Vieira, Stéphane Mouly, Abdellatif Tazi, Julien Haroche, Trecy Goncalves, Damien Sène, Jean-François Emile, Cloé Comarmond

**Affiliations:** ^1^ Department of Internal Medicine, Lariboisière Hospital, Assistance Publique des hôpitaux de Paris (APHP), Université Paris Cité, Paris, France; ^2^ Department of Cardiology, Lariboisière Hospital, Université Paris Cité, Paris, France; ^3^ Department of Pulmonology, Saint-Louis Hospital, Université Paris Cité, Paris, France; ^4^ Department of Neurology, Lariboisière Hospital, Université Paris Cité, Paris, France; ^5^ Department of Pathology, Institut Mutualiste Montsouris, Paris, France; ^6^ Department of Pulmonology, Institut Mutualiste Montsouris, Paris, France; ^7^ Department of Internal Medicine and Clinical Immunology, Pitié-Salpêtrière Hospital, Sorbonne Université, Paris, France; ^8^ Pathology Department, Ambroise Paré Hospital, Assistance Publique des Hôpitaux de Paris (APHP), Paris, France

**Keywords:** Erdheim-Chester disease, pleural thickening, periaortitis, heary kidney, pericarditis

## Abstract

Erdheim–Chester disease (ECD) is a rare histiocytic disorder with localized presentations or multisystem disease. Clinical presentations of ECD are usually non-specific and depends on the site of involvement. ECD can involve one or several organs. Clinical manifestations range from asymptomatic lesions to severe and life-threatening organ dysfunction. Hence, accurate and timely diagnosis is challenging given the rarity and varied presentation of ECD. The most common clinical manifestations are bone pain related to osteosclerosis, usually in the lower limbs. We report here a case with no obvious clinical manifestation of ECD preceding initial recurrent pleural effusions. The diagnosis of ECD was suggested based on pleural thickening revealed by relapsing pleural effusions combined with radiological finding of a coated aorta and slight perirenal infiltrate. Pleural biopsy revealed collagen fibrosis, and immunohistochemistry with the anti-CD163 antibody showed an important infiltration by histiocytes, strong cytoplasmic phosphorylated ERK in the lesional cells, and positive factor XIIIa staining. A cell-free DNA from peripheral blood revealed negative *BRAF* mutation and the presence of *MAP2K1* mutation, a key driver mutation in ECD. The diagnosis is often suggested based on clinic-radiological presentation but requiring histopathology to establish a final diagnosis of ECD. Plasma cell-free DNA is a promising and non-invasive tool to detect key driver mutations.

## Introduction

A 75-year-old smoking man was admitted in October 2023 to our internal medicine department for relapsing pleural effusions during the last 3 months. He presented no prior comorbidity except 6 months before (in February 2023) when his wife found him on the ground with impaired consciousness. The diagnosis of ischemic stroke related to atherothrombosis was then made, and he underwent thrombolysis followed by medical treatment.

Two months after the stroke, he presented thoracic pain and dyspnoea for which he was admitted to the emergency hospital where the diagnosis of myocardial infarction or pulmonary embolism was ruled out. Thoracic and supraortic computed tomography scan found bilateral pleural infusion. The pleural puncture revealed sero-sanguineous fluid with 50% neutrophiles, 36% lymphocytes no tumoral cell, total proteins 30.3 g/L, and LDH 849 U/L and negative culture. Since the patient was on dual antiplatelet drugs, the diagnosis of an iatrogenic hemothorax was first suspected and Clopidogrel was thus stopped but pleural effusion still recurred with the appearance of pleural thickening. A retrospective analysis of aortic and supraortic computed tomography scan performed at the time of stroke revealed the existence of a discrete filling of the pleura even before he had undergone thrombolysis therapy. He was then admitted in internal medicine for further investigation.

**Figure 1:**
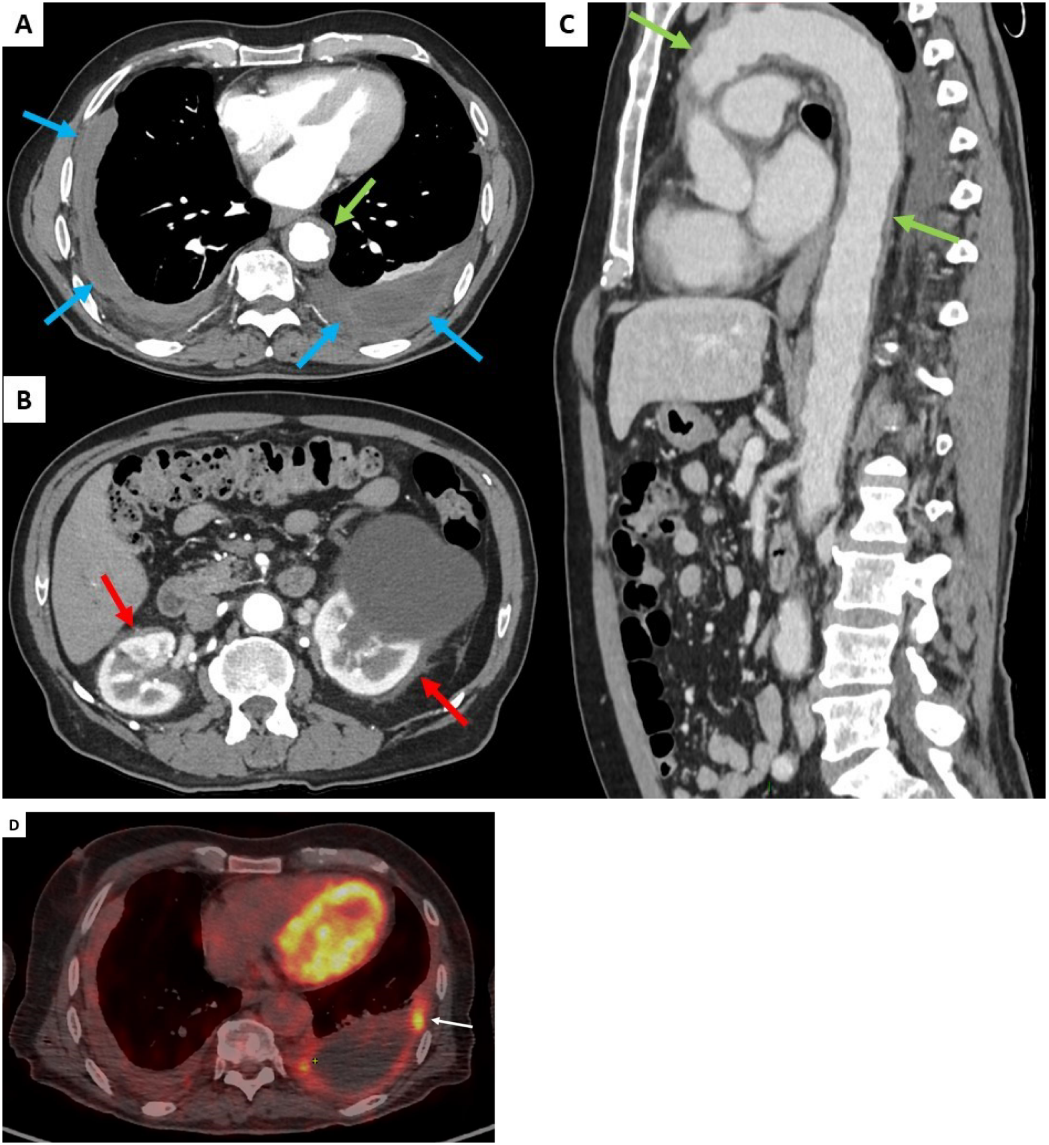
Contrast-enhanced thoracic and abdominal CT-scan transverse sections (blue arrows, **A**), symmetrical irregular soft-tissue infiltration in the perirenal spaces (red arrows, **B**), and thickening of the aorta (green arrows, **C**). FDG uptake was found along the left pleura (white arrow, **D**).

On admission, physical examination showed normal results including cardiovascular and chest auscultation. He denied weight loss, chest pain, and dyspnoea. Laboratory results showed normal C-reactive protein (4 mg/L), negative IGRA test (Quantiferon^®^), normal IgG4 serum levels, negative ANCA, and negative syphilis serology. Brain gadolinium-enhanced magnetic resonance imaging (MRI) revealed ischemic lacunar sequalae of the left corona radiata and left superior cortex, without intracranial periarterial infiltration. Chest and abdominal computed tomography (CT) scan results showed bilateral pleural thickening (**Figure 1A**), symmetrical irregular perirenal infiltration (**Figure 1B**), and periaortic sheathing of the thoracic and abdominal aorta (**Figure 1C**). An F - 18 fluorodesoxyglucose (FDG)-positron emission tomography (PET) results showed strong FDG uptake found along the left pleura (left pleura SUVmax = maximum standardized uptake value = 6.2), moderate uptake in thoracic and abdominal aortas (arch of aorta SUVmax = 3.9 and abdominal aorta SUVmax = 2.9), and no bone hypermetabolism (**Figure 1D**).

**Figure 2:**
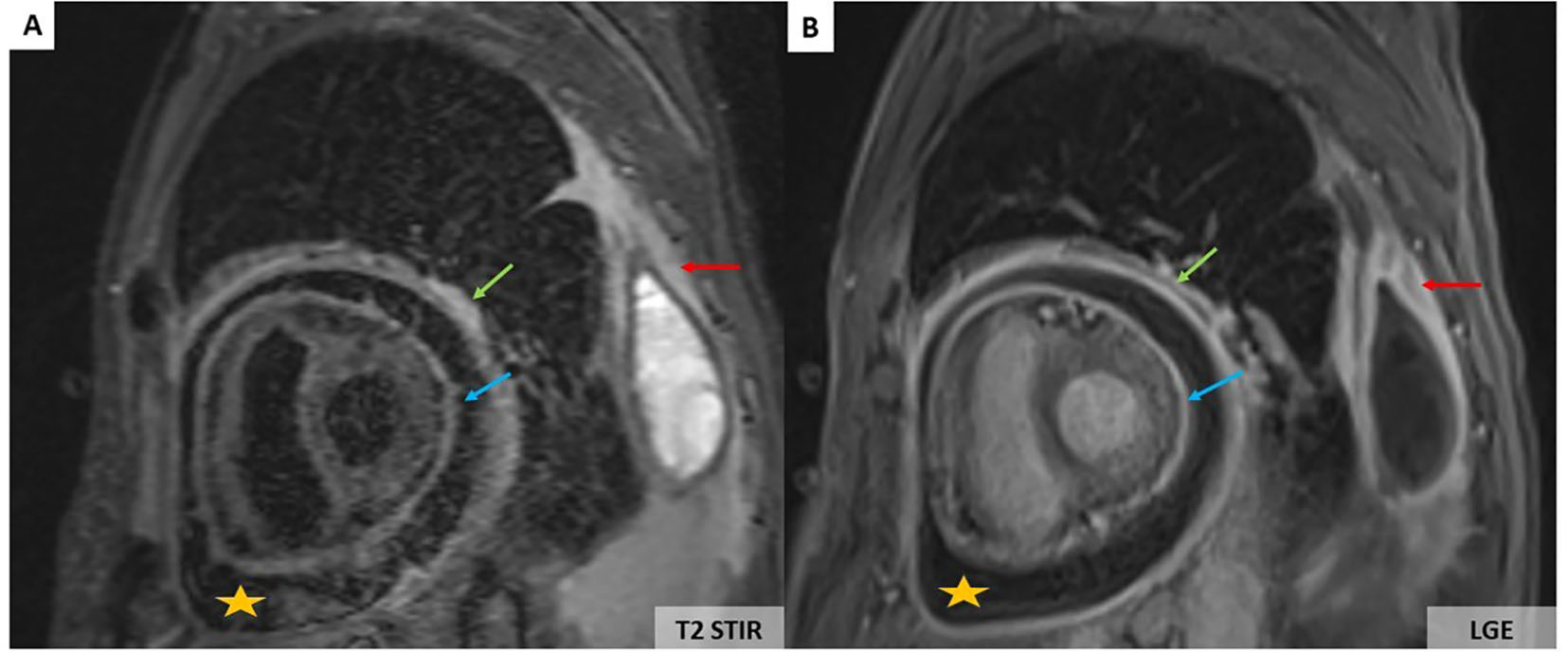
CMR showing large pericardial effusion (orange stars) associated with thickening of the pericardium **(A, B)**. On the T2-weighted short-tau inversion recovery (STIR) sequence, there is an increase in signal of the parietal (green arrows) and visceral pericardium (blue arrows) suggesting recent pericardial inflammation **(A)**. Pericardial hyperenhancement of the thickened parietal and visceral pericardium secondary to pericardial inflammation associated with left pleural thickening (red arrows) on the late gadolinium enhancement sequence **(B)**.

However, a progressive dyspnoea appeared a month later (November 2024) leading to performing a transthoracic echocardiography which revealed a moderate pericardial effusion without echocardiographic signs of cardiac tamponade. The troponin level was normal, and C-reactive protein values increased up to 12 mg/L. The patient received a 2-week course of non-steroidal anti-inflammatory drugs in combination with acetaminophen. His clinical status did not improve, and the C-reactive protein level further increased to 110 mg/L. Cardiovascular magnetic resonance (CMR) imaging showed large pericardial effusion associated with thickened pericardium (**Figures 2A, B**).

**Figure 3:**
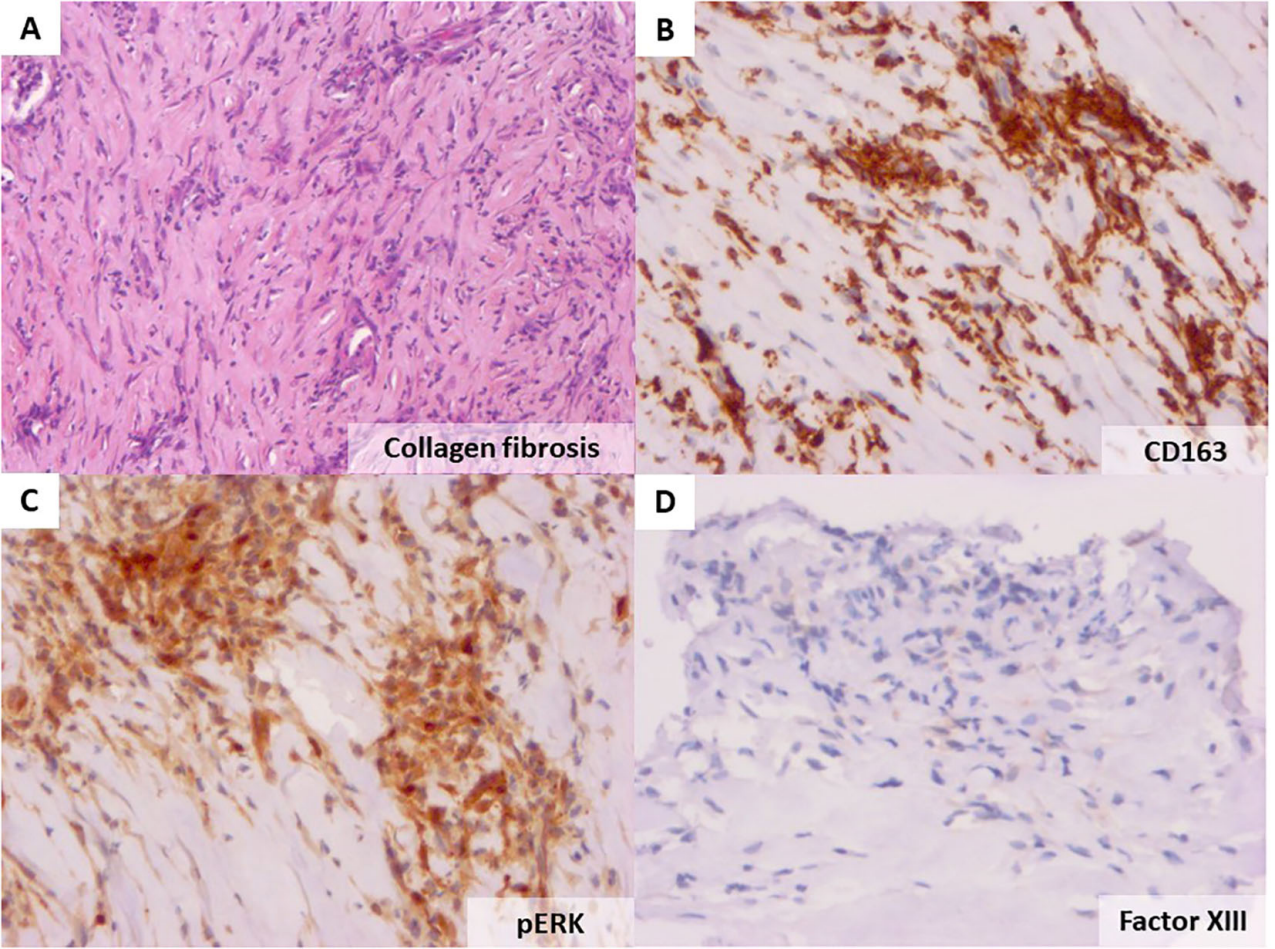
Haematoxylin and eosin staining of the patient’s pleural biopsy showing collagen fibrosis (original magnification ×40, **A**). Immunohistochemistry with the anti-CD163 antibody showing an important infiltration by histiocytes (×20, **B**), strong cytoplasmic phosphorylated ERK in the lesional cells **(C)**, and positive factor XIIIa staining **(D)**.

**Figure 4:**
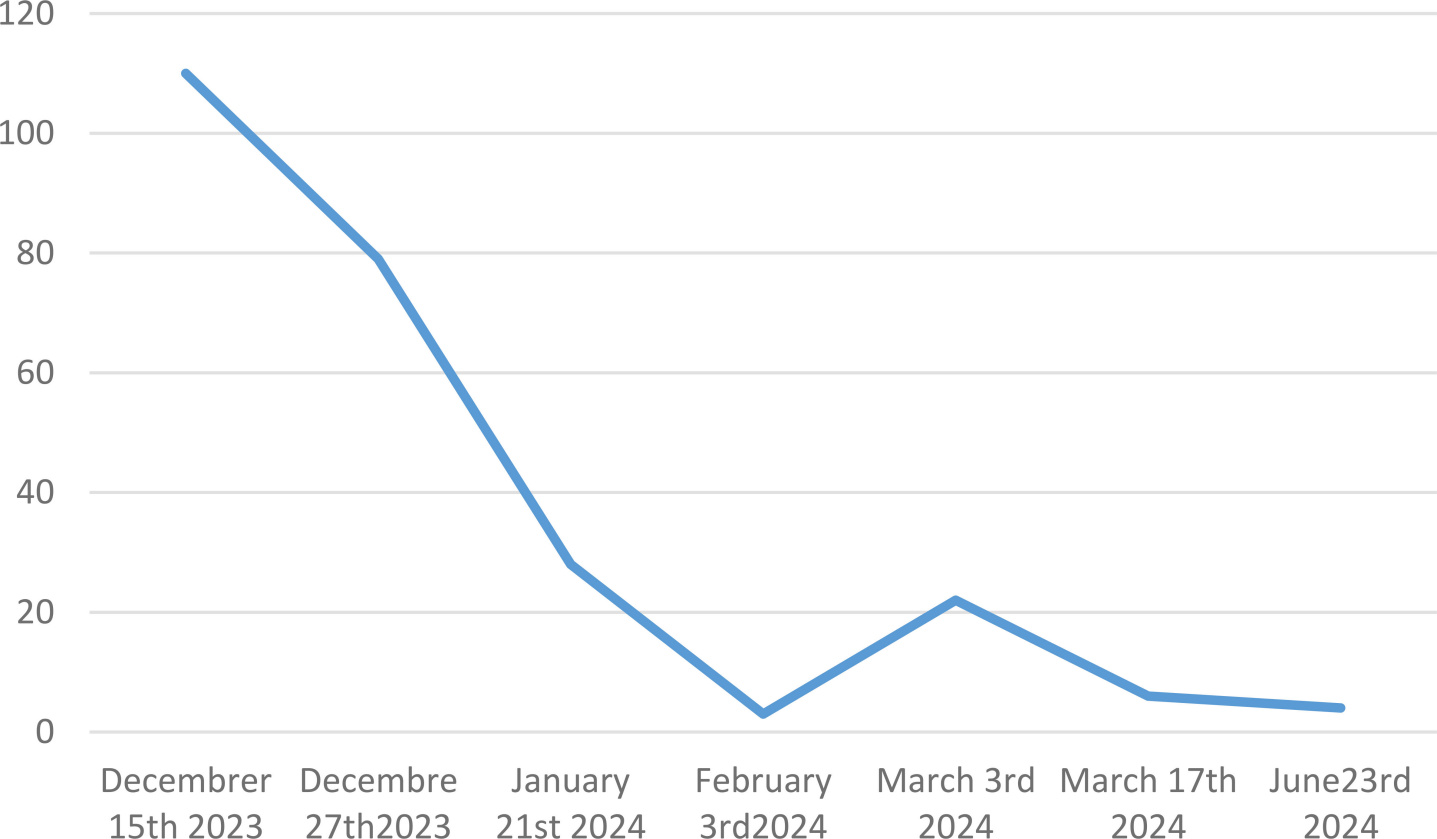
C reactive protein monitoring values from baseline at diagnosis through 6 months of treatment with cobimetinib.

**Figure 5:**
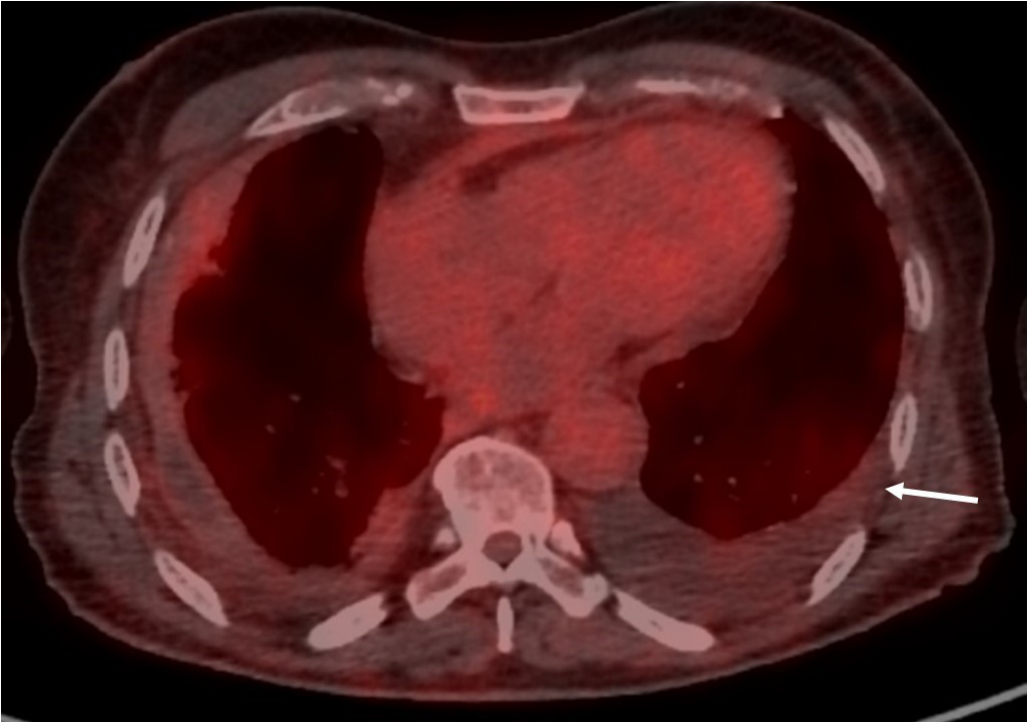
FDG uptake regression along the left pleura (white arrow) 1 year after diagnosis and treatment introduction.

To address the diagnosis of these pleural and pericardial effusions and thickening of the aorta, we planned pleural biopsy, guided by the most FDG-avid site and the most easily accessible biopsy site. An ultrasound-guided core needle (18G) pleural biopsy was performed by a pulmonologist. Histopathologic findings showed significant non-specific collagen fibrosis without granuloma (**Figure 3A**). A polymerase chain reaction test for tuberculosis showed negative results on pleural tissue. Immunohistochemistry revealed a strong and diffuse expression of CD163 by histiocytes (**Figure 3B**), phosphorylated ERK (**Figure 3C**), positive factor XIIIa (**Figure 3D**), and negative CD1a. *BRAF-V600E* mutation was not detected with molecular analysis of pleural tissue. As the pleural tissue sample was insufficient for molecular analysis with targeted-capture next-generation sequencing (NGS), cell-free DNA from peripheral blood revealed negative *BRAF* mutation and the presence of *MAP2K1* mutation. Empiric treatment with the MEK inhibitor was initiated (cobimetinib 60 mg daily) when diagnosis was made. After 1 month of treatment, C-reactive protein (CRP) values consistently decreased from 110 mg/L at baseline of diagnosis to complete normalization 1 month later. Two months later, the CRP values were quite normal (4mg/L) (**Figure 4**) but a little raise was noticed in March 2024 after 3 months of therapy due to ORL infection. Other side effects were also observed like acneiform lesions and diarrhoea which required a reduction in doses of cobimetinib to 40 mg/L with the same disease control. Pericardial effusion disappeared on transthoracic echocardiography. CT scan results showed persistent periaortic sheathing of the thoracic and abdominal aortas, bilateral pleural thickening, and perirenal infiltration. However, FDG-PET-CT performed 1 year after diagnosis and cobimetinib introduction showed complete regression of uptakes in the pleura and aorta (**Figure 5**). The diagnosis of Erdheim–Chester disease (ECD) should be raised in a compatible clinical, radiological, histopathological, and molecular setting. For ECD patients without BRAF-V600E mutation, empiric treatment with the MEK inhibitor should be strongly considered as first-line therapy.

## Discussion

Erdheim–Chester disease (ECD) is a rare histiocytic disorder with localized presentations or multisystem disease. Clinical manifestations of ECD are usually non-specific and depends on the site of involvement (1). ECD can involve single or multiple organs, and presentations range from asymptomatic lesions detected incidentally on imaging to severe and life-threatening organ dysfunction (**Table 1**). Hence, accurate and timely diagnosis is challenging given the rarity and varied presentation of ECD. The most common clinical manifestations are bone pain related to osteosclerosis, usually in the lower limbs (2). Other systemic manifestations include cardiac mass and periaortic involvement; diabetes insipidus; retro-orbital infiltration; and retroperitoneal, lung, central nervous system (CNS), skin, and periorbital xanthelasma, usually in combination (**Table 1**) (2). Common constitutional symptoms including fever, weight loss, and night sweats might be mistaken for tuberculosis. In our case, it is because no obvious clinical manifestation of ECD preceded initial acute ischemic stroke and pleural effusions. Diagnosis was suggested based on pleural thickening revealed by relapsing pleural effusions combined with radiological finding of a coated aorta and slight perirenal infiltrate. Albeit unusual neurological manifestation of ECD, ischemic stroke has been reported related to sheathing of intracranial vessels (3). Lung involvement in ECD is mostly asymptomatic or patients may have dry cough and dyspnoea related to either lung parenchyma or pleural lesions. In our case, pleural lesions that were already noticed in aortic computed tomography scan before thrombolysis confirmed that pleural involvement was maybe the first symptom to appear. Some authors also reported pleural thickening during ECD, but pleural involvement was not histologically proved. Kherabi (4) related the existence of ECD with pleural involvement in the form of recurrent pleuro-pneumopathy in a 76-year-old woman. Bronchoalveolar lavage fluid was sterile with no alveolitis. In that case, the diagnosis of ECD was suspected because of the history of chronic myelomonocytic leukaemia, a coated aorta, a hairy kidney aspect, and a pseudotumoral infiltration of the atrioventricular groove. Pleural biopsy was not performed, but perirenal fat biopsies confirmed the diagnosis with mutation of BRAF and NRAS. Cobimetinib therapy led to complete regression of pleural effusion on CT scan and other symptoms. A case report of a 71-year-old woman in China (5) who complained of recurrent fever, chest tightness with pleural effusion, and atelectasy was noted. Pleural puncture revealed few mesothelial cells and histiocytes without atypical cells. The diagnosis was made with bone marrow biopsy. In our context, only pleural biopsy was performed as this was the most accessible site. Immunochemistry was compatible with non-Langerhans cell histiocytis by diffuse expression of CD163, pERK, and negative CD1a. Regarding aetiologies of non-Langerhans cell histiocytosis, the absence of lymphadenopathy made the diagnosis Rosai–Dorfman disease unlikely. Therefore, in context of non-Langerhans cell histiocytis, the “hairy kidney” appearance in the CT scan associated with periaortic involvement and pleuropericardial effusions were more compatible with Erdheim–Chester disease. The fact that NGS was performed on peripheral blood instead of the pleural tissue sample is one weakness of our study as the presence of MAP2K1 mutation was not confirmed on pleural tissue. Pleural involvement has been reported in 15%–40% of ECD patients with unilateral or bilateral pleural effusion and focal or diffuse thickening of the visceral pleura (6). Unlike pulmonary Langerhans cell histiocytosis (LCH), no association with cigarette smoking has been reported. Treatment is indicated for most ECD patients, except some cases of asymptomatic minimal burden disease, which can be monitored closely (1). Currently, treatment includes BRAF and MEK inhibitors, interferon-α, and rarely cladribine (**Table 1**).

**Table 1:** Clinical and radiological characteristics, and treatment strategies according to histiocytic disorders.

Characteristics	Erdheim–Chester disease (ECD)	Langerhans cells histiocytosis (LCH)	Rosai–Dorfman–Destombes disease (RDD)
Age/gender	Adult Incidence M>F	Childhood or adult	Childhood, or young adult Incidence F>M
Clinical manifestations	Long/lower-extremity bone pain (50%) Diabetes insipidus (45%) Hypogonadism (45%) Periorbital xanthelasma (30%) Orbital masses and exophthalmos (25%) Renal insufficiency from ureteral obstruction Pericardial disease, tamponade (<10%)	Osteolytic lesions Lymphadenopathy Dark red or brown skin lesions Pulmonary involvement (40 - 50%), with pneumothorax (10%) Central diabetes insipidus	Bone pain (pathologic fractures rare) Massive lymphadenopathy Reddish-brown or yellow skin lesions Intrathoracic manifestations (<5%), usually with concurrent lymphadenopathy Hematologic manifestations: anaemia (67%), leucocytosis with neutrophilia (60%)/eosinophilia, thrombocytopenia, hypergammaglobulinemia
Imaging features	Meta-diaphyseal osteosclerosis (95%) Perinephric infiltrates, retroperitoneal fibrosis, “hairy kidneys” (65%) Periaortic sheathing “coated aorta”(60%) Pleural thickening/effusions, interlobular septal thickening, ground-glass opacities, centrilobular opacities (50%) Mediastinal infiltration, right atrial pseudotumor (40%) CNS infiltration (parenchymal or dural mass, hypothalamic or pituitary stalk infiltration 40%)	Upper lobe predominant nodular and cystic lung lesions in a smoker Punched-out lytic osseous lesions, often involving flat bones (skull, sternum, ribs, pelvis)	Lymphadenopathy Pseudotumor Meta-diaphyseal osteolytic or mixed lytic/sclerotic lesions ± soft tissue extension
Treatment strategy according to the presence of BRAF mutation and critical organ involvement	• Presence of BRAF mutation BRAF inhibitor (vemurafenib or dabrafenib) ± systemic corticosteroids, if oedema or acute symptoms • Absence of BRAF mutation *If cardiac/neurologic disease or end-organ dysfunction:* MEK inhibitor (cobimetinib) *If low-burden disease involving bones and retroperitoneum*: Cytokine-directed therapy (anakinra) ± systemic corticosteroids, if oedema or acute symptoms • No access to BRAF or MEK inhibitors *If high-burden disease*: IFN-a/PEG–IFN-a or Cladribine (rare) ± systemic corticosteroids, if oedema or acute symptoms	• Critical organ involvement *If presence of BRAF mutation:* BRAF inhibitor (vemurafenib or dabrafenib) *If absence of BRAF mutation:* Chemotherapy (vinblastine, cladribine) or MEK inhibitor (cobimetinib) • No critical organ involvement *If single-system pulmonary LCH:* Limited-disease: smoking cessation PD: systemic (cladribine) or targeted therapy *If bone-only disease:* Bisphosphonates, oral MTX, hydroxyurea Chemotherapy, if ≥3 lesions or PD *If skin-only disease:* Topical therapy, oral MTX, 6-MP, Thalidomide or lenalidomide Chemotherapy, if PD *If multi-system LCH:* Chemotherapy • Relapsed/refractory LCH Alternate chemotherapy agent or BRAF inhibitor, if *BRAF* mutation or MEK inhibitor	• Nodal/cutaneous RDD *If symptomatic single-site disease:* Biopsy/resection *If unresectable or multifocal disease*: Systemic therapy • Extranodal RDD *If neurologic or end-organ dysfunction:* Resection/debulking of sites *If unresectable or multifocal disease*: Systemic therapy • Relapsed/refractory/severe RDD MEK inhibitor, if driver mutation identified with next-generation sequencing for MAPK pathway mutations (e.g., KRAS, MAP2K1)

6-MP, 6-mercaptopurine; MTX, methotrexate; PD, progressive-disease.

Imaging findings are paramount to establishing the diagnosis of ECD. Imaging is used to ascertain the sites of involvement, delineate the extent, and monitor evolution during follow-up of this systemic disease. Given the multisystemic involvement, a wide array of radiological modalities is needed. At baseline evaluation, full-body (skull-to-toes) FDG PET-CT imaging including distal extremities, brain gadolinium enhanced MRI, and cardiac MRI in all newly diagnosed patients is useful to identify disease burden including clinically occult lesions (1). FDG PET-CT performed prior to biopsy can be an important tool for guiding biopsy targets, preferentially in the most FDG-avid sites that are accessible and safe. Imaging of involved disease sites can be useful for response assessment and monitoring (1). A regression of infiltration on imaging is significantly associated with improved survival (7).

Some of the most striking radiological signs of ECD are the long bone bilateral cortical sclerosis (80%-95%) with bilateral symmetric osteosclerosis of meta-diaphysis of femur, tibia, and fibula (pathognomonic), as well as the hairy kidney appearance on CT scan (63%), the coated aorta (40%), and the right atrium pseudo-tumoral infiltration (36%) (8). Bone scan usually displays bilateral and symmetric cortical osteosclerosis of the long bones. FDG PET-CT for early evaluation of whole-body skeletal metabolism shows frequently evidence of symmetric and abnormally strong labelling of the distal ends of the long bones of the lower limbs (and sometimes the upper limbs) (7). It is often pathognomonic in concert with other findings. Our patient has no bone pain or any skeletal involvement.

Up to 50% of patients show lung and pleural involvement on the CT scan (5). Lung findings in ECD include reticular infiltrative opacities, peribronchovascular infiltrate, focal or diffuse smooth interlobular septal thickening, multifocal micronodules, and ground-glass opacities (8). Honeycombing is rare (<10%) (9). Pleural ECD thickening can be focal or diffuse, and unilateral or bilateral (**Figure 1A**) (5). No specific pattern or topography characterizes infiltrative lung disease of ECD. The presence of lung lesions and pleural thickening in combination with other systemic findings suggests the diagnosis.

The most common extraosseous site affected in ECD is retroperitoneum including the kidneys, involved in approximately 68% of cases, remaining asymptomatic, or revealed by abdominal pain or dysuria (2, 6, 7). Perirenal infiltration, named the “hairy kidney sign,” may cause large bilateral soft tissue masses and compression effect on kidney (**Figure 1B**) (7). Retroperitoneal fibrosis is the major differential diagnosis.

Vascular involvement affects mainly the aorta with adventitial histiocytic infiltration and periarterial fibrosis, observed in 56%–85% of patients (2, 6, 7). Diffuse and circumferential infiltration of aorta is characterized by “coated aorta” appearance, observed in 23%–30% of patients (**Figure 1C**). In our case with initial ischemic stroke, differential diagnosis could also be made with large vessel vasculitis (giant cell arteritis, GCA) where inflammation on the aorta potentially led to FDG uptake. Unlike GCA, aortic infiltration could not resolve during follow-up and may be associated with fibrotic or atherosclerotic lesions for which ECD treatments have no substantial effects (10).

Pericardium, myocardium, and coronary arteries lesions are observed in up to 40%–70% of ECD patients (10). Clinical presentations may be severe with worse prognosis. Arrhythmias, myocardial ischaemia, valvular dysfunction, and heart failure are more common in older patients (10). Cardiac MRI performed at baseline can identify cardiac involvement and evaluate the extent of ECD (10). The pericardium may be thickened with effusion that can cause cardiac tamponade (**Figures 2A, B**). However, massive pericardial effusions are rare (10). Myocardial infiltration usually involves the right atrium and right atrioventricular groove. Myocardial involvement is best seen on MRI as T1W hypointense focal lesions with post-contrast enhancement. Infiltration of coronary arteries is reported in 23% of patients, most commonly the right coronary artery, and can lead to ischemic cardiomyopathy (10).

The histopathologic diagnosis of ECD often shows non-specific inflammatory and fibrotic results. Histopathology of the affected tissues shows infiltration by foamy histiocytes surrounded by fibrosis with or without the presence of Touton giant cells (1). The main pathologic features of histiocytic disorders are summarized in **Table 2** (1). In ECD lesions, CD68, CD163, and Factor XIII stainings are positive, with CD1a and Langerin testing being negative (2, 6, 7). A biopsy is often performed even if clinical and imaging features are suggestive of EDC to also establish *BRAF* and MAPK-ERK pathway mutational status (1, 11). Mutations activating the MAPK pathway are presented in 80% of ECD (*BRAF V600E* in 57%-70% of cases, and *MAP2K1* in 20%). Like in our case, 40% of *BRAF V600E* wild-type ECD patients will harbour a mutation in *MAP2K1*. The BRAFV600E mutation is associated with lung and pleural involvements and more affected organs (6). Mutations affecting other signalling molecules (e.g., *NRAS*, *KRAS*) may also be found. Negative BRAF results could be confirmed with more than one test modality or more than one sampled site, especially in patients without osseous involvement (1). However, obtaining enough tissue for BRAF testing can be challenging, given tissue with a high ratio of fibrosis to histiocytes. In case of insufficient cellularity in the specimen to conduct molecular analysis, a properly validated BRAF-VE1 or phosphorylated ERK stain may help if there is moderate to strong cytoplasmic staining in the lesional cells (**Figure 3C**). Interestingly, circulating cell-free DNA (cfDNA) is also able to detect key driver mutation for patients in whom biopsy of lesional tissue is difficult to obtain or with insufficient tissue samples to perform molecular analysis (11). In cases like ours for which a tissue specimen is insufficient for molecular analysis, cfDNA testing is a reasonable alternative (1, 11). ECD with negative *BRAF* may be treated with MEK inhibitors.

**Table 2:** Histopathologic features of the histiocytic disorders.

Feature	Erdheim–Chester disease	Langerhans cells histiocytosis	Rosai–Dorfman–Destombes disease
Pathological features			
Xanthomatous histiocytes Touton giant cells Emperipolesis	Yes Yes, mainly in skin tissue Rare	No No No	No No Abundant
Immunophenotype			
CD163 (surface) CD1a (surface) PS100 (cytoplasmic/nuclear) CD207/langerin	Yes No Variable: negative or weak positive, + in 20 - 30% of ECD cases No	No, expressed in a minority <10% Yes Yes Yes	Yes, negative in a minority <10% No Yes No
Molecular features			
*BRAF V600E* *MAP2K1* RAS isoforms (*KRAS*, *NRAS*)	50% 20% <10%	55% 15% <5%	<5% 15% 30%
**Histopathologic features**	Xanthomatous histiocytes with small nuclei and surrounding fibrosis CD163+CD68+ CD1a− PS100± BRAF or MAPK-ERK pathway mutations Touton giant cells ±	Histiocytes with elongated, grooved nuclei, ± with intermixed eosinophils CD68+ CD1a+ PS100+ BRAF V600E mutation Birbeck granule	Histiocytes with vesicular nucleus and abundant clear cytoplasm CD68+ CD1a− PS100+ Emperipolesis

Emperipolesis = intracytoplasmic inflammatory cells including plasma cells and lymphocytes.

BRAF V600E, B-Raf proto oncogene, mutation V600E.

MAP2K1, mitogen-activated protein kinase kinase 1.

KRAS, V-Ki-ras2 Kirsten rat sarcoma viral oncogene.

NRAS, neuroblastoma RAS viral oncogene.

## Conclusion

- The diagnosis of ECD is a multidisciplinary challenge where imaging plays a crucial role to assess disease burden and guide biopsy target and follow-up.
- Pleural thickening may be a manifestation of ECD, even more so combined with multiorgan coating.
- While the final diagnosis is established by histopathology, the initial diagnosis is often suggested based on clinic-radiological presentation, and plasma cell-free DNA is a promising and non-invasive tool to detect key driver mutations.
- Cobimetinib is the reference therapy for ECD without BRAF mutation.

## Data Availability

The raw data supporting the conclusions of this article will be made available by the authors, without undue reservation.
